# Novel Coronavirus in Cape Town Informal Settlements: Feasibility of Using Informal Dwelling Outlines to Identify High Risk Areas for COVID-19 Transmission From A Social Distancing Perspective

**DOI:** 10.2196/18844

**Published:** 2020-04-06

**Authors:** Lesley Gibson, David Rush

**Affiliations:** 1 School of Engineering University of Edinburgh Edinburgh United Kingdom

**Keywords:** COVID-19, Cape Town, informal settlements, social distancing, GIS, pandemic, outbreak, infectious disease, public health, geographic data, risk

## Abstract

**Background:**

The challenges faced by the Global South during the coronavirus disease (COVID-19) pandemic are compounded by the presence of informal settlements, which are typically densely populated and lacking in formalized sanitation infrastructure. Social distancing measures in informal settlements may be difficult to implement due to the density and layout of settlements. This study measures the distance between dwellings in informal settlements in Cape Town to identify the risk of COVID-19 transmission.

**Objective:**

The aim of this paper is to determine if social distancing measures are achievable in informal settlements in Cape Town, using two settlements as an example. We will first examine the distance between dwellings and their first, second, and third nearest neighbors and then identify clusters of dwellings in which residents would be unable to effectively practice social isolation due to the close proximity of their homes.

**Methods:**

Dwellings in the settlements of Masiphumelele and Klipfontein Glebe were extracted from a geographic information system data set of outlines of all informal dwellings in Cape Town. The distance to each dwelling’s first, second, and third nearest neighbors was calculated for each settlement. A social distance measure of 2 m was used (buffer of 1 m, as dwellings less than 2 m apart are joined) to identify clusters of dwellings that are unable to effectively practice social distancing in each settlement.

**Results:**

The distance to each dwelling’s first 3 nearest neighbors illustrates that the settlement of Masiphumelele is constructed in a denser fashion as compared to the Klipfontein Glebe settlement. This implies that implementing social distancing will likely be more challenging in Masiphumelele than in Klipfontein Glebe. However, using a 2-m social distancing measure, it was demonstrated that large portions of Klipfontein Glebe would also be unable to effectively implement social distancing.

**Conclusions:**

Effectively implementing social distancing may be a challenge in informal settlements due to their density. This paper uses dwelling outlines for informal settlements in the city of Cape Town to demonstrate that with a 2 m measure, effective social distancing will be challenging.

## Introduction

The World Health Organization (WHO) declared the outbreak of the novel coronavirus disease (COVID-19) to be a pandemic on March 11, 2020, with the WHO director-general stating, “This is not just a public health crisis, this is a crisis that will touch every sector. So every sector and every individual must be involved in the fights,” [1]. In the absence of a vaccine, tools such as isolation and quarantine, social distancing, and community containment become vital in preventing the person-to-person spread of disease by separating people to interrupt transmission [2]. COVID-19 statistics for South Africa show that, as of March 30, 2020, there were 1326 cases in South Africa with 324 reported cases in the Western Cape Province with a total of 3 deaths in the country [3]. In response to the COVID-19 pandemic, President Cyril Ramaphosa announced a nationwide lockdown for 21 days effective at midnight on March 26, 2020 [4]. This paper was first published online prior to this announcement; however, the method and results can be interpreted in light of the lockdown decision and can be used to guide any easing of restrictions going forward.

The challenges faced by the developing world during the pandemic are compounded by the presence of informal settlements, which are typified by being densely populated and lacking formalized sanitation infrastructure [5]. Cape Town, the capital city of the Western Cape Province with an estimated population of 4 million, is South Africa’s second most populous city after Johannesburg and Africa’s 10th most populous city [6]. Cape Town has not been immune to the rise of urbanization, and migration to the city has led to the establishment of many informal settlements. Housing in informal settlements is unregulated by the state, planned by local communities, and typically constructed in a haphazard fashion using cheap and recycled building materials. Due to competition for space, homes can be built close together with only narrow access paths. Some settlements are less densely constructed, but these tend to be the newer settlements located further away from the city in locations where there is little economic opportunity. There are an estimated 146,000 households living in informal settlements in Cape Town, many of which are not recognized as permanent, with the residents lacking occupation rights and security of tenure [7]. It is further estimated that only one-third of the toilets in Cape Town’s informal settlements are permanent infrastructure with the rest being temporary toilets that are provided and cleaned by private companies [7]. Other challenges include the particularly high HIV burden borne by residents of informal settlements in comparison to other settlement types [8]. Although there is no evidence that the risk of infection or complications of COVID-19 are different amongst people living with HIV when compared with the general population, people living with advanced HIV disease and who are not taking antiretroviral treatment are at an increased risk of infections, in general [9].

Social distancing aims to reduce the interactions between people in a broader community and is useful for communities where individuals may be infectious but have not yet been identified and are thus not isolated [2]. Furthermore, it has been proposed that social distancing be implemented in a rationally layered manner to protect individuals with a higher risk of mortality [10]. Should social distancing measures not be effective, the next stage, known as community containment, may need to be implemented. This involves reduction of personal interaction at the community level, which is ethically more challenging, and its implementation requires close partnership and cooperation with law enforcement [2].

Given the nature of informal settlements, if social distancing is implemented by the state, it should be established whether an individual urban settlement is able to achieve this based on the layout of the particular settlement. Due to the density of dwellings in informal settlements, effectively implementing social distancing may be a challenge. The objective of this paper is to determine if social distancing measures are achievable in informal settlements in Cape Town using two settlements as an example. First, we examined the distance between dwellings and their first, second, and third nearest neighbors. Second, we identified clusters of dwellings in which residents would be unable to effectively practice social isolation due to the close proximity of their homes. It should be noted that this study is based solely on one data set (the outline of informal dwellings), and it is envisaged that public health scientists could incorporate this data set as one of many parameters in specific risk modelling, should it prove useful. Vulnerability mapping of COVID-19 in the South African context, which considers factors other than distances between dwellings, has been written for the Gauteng Province [11] and could similarly be applied elsewhere in South Africa.

## Methods

This paper looks at the feasibility of social distancing in two informal settlements in Cape Town as an effective measure to prevent transmission of COVID‑19 in these urban environments. In particular, it looks at the layout of the settlements with respect to the distance between dwellings and their nearest neighbors to determine if a social distancing approach is feasible in these environments. The assumption was made that all outer boundaries of a dwelling are a potential zone of transmission. It is likely that the risk of transmission will be higher at openings such as doors and windows; however, in the absence of these data, all boundaries of a dwelling were treated equally.

**Figure 1:**
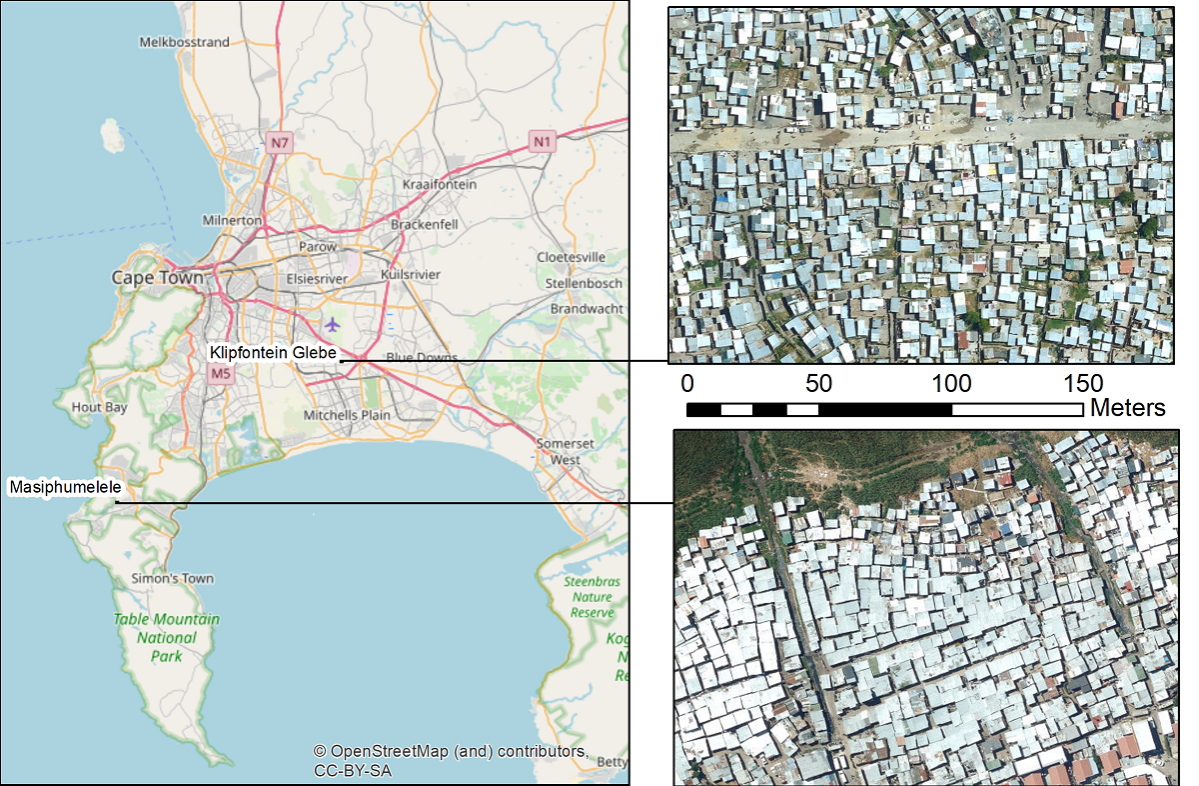
City of Cape Town and the location of Masiphumelele and Klipfontein Glebe.

Two informal settlements in Cape Town have been selected to demonstrate the application. The location of these two settlements and zoomed in aerial photography of typical areas within the settlements are shown in Figure 1. It has been widely reported that settlements are typically overpopulated with a high dwelling density, but quantitative data on this has been lacking until now. Roof outlines of all informal dwellings in informal settlements in the city of Cape Town have been mapped from aerial photography captured in February 2018 [12] and can be used to obtain data on the distance between a dwelling and the nearest neighbors.

The dwelling outlines are in the form of a geographic information system (GIS) vector data set (shapefile), with individual polygons representing either individual dwellings or, in cases where dwellings are built so close to each other that they cannot be visually separated, clusters of connected dwellings. Working within the GIS software ArcGIS 10.5.1 (Esri), dwellings corresponding to each settlement were selected and saved into separate shapefiles. The proximity tool “Generate Near Table” was then used to calculate the distance to each dwelling’s first, second, and third nearest neighbors. Subsequently, in Microsoft Excel, the normalized distribution of these distances was calculated for the first, second, and third nearest neighbor in each settlement to provide an overview of the density of each settlement in relation to social distance measures.

**Figure 2:**
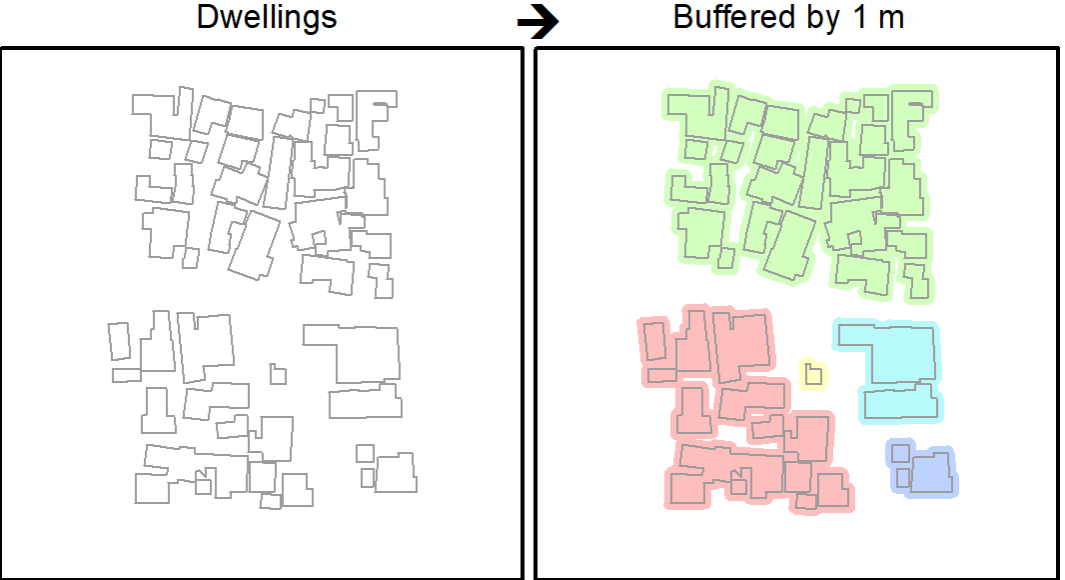
Illustration of how self-isolating clusters of dwellings are identified.

The UK guidelines on social distancing state that if a person meets another while outdoors, they should ensure a 2-m distance between them [13]. If such a measure was to be implemented in Cape Town, using the dwelling data set, it is possible to identify clusters of homes that are unable to effectively self-isolate due to their proximity to neighbors (ie, a person who leaves their home will immediately be within 2 m of another home and may either spread or become infected by the virus). This would be exacerbated if more than one person in a cluster was outside their home at the same time. Taking the dwellings’ outline data set in ArcGIS 10.5.1, the proximity tool “Buffer” was used to expand the outline of individual dwellings by 1 m. Thus, if two dwellings are within 2 m of each other, their buffers intersect and a single polygon grouping of these dwellings is drawn. This is illustrated in Figure 2 where each color represents the groupings of dwellings that would have to self-isolate together, as individual self-isolation would likely be ineffective.

## Results

**Figure 3:**
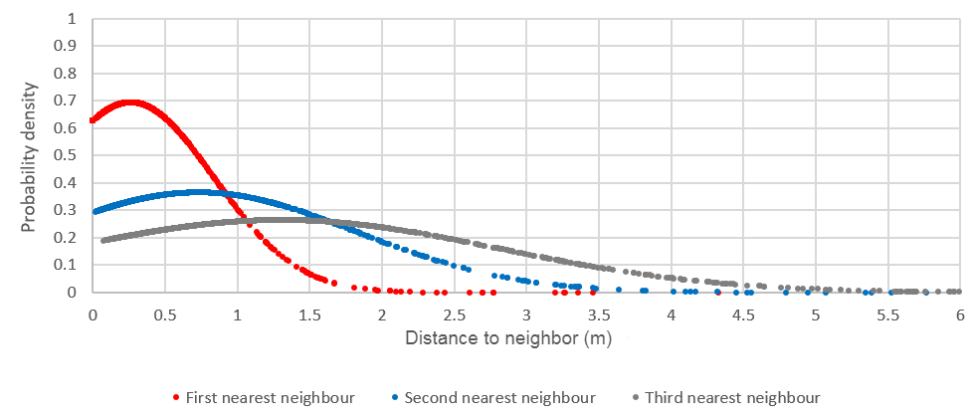
Normalized distribution of the distance between dwellings and their first, second, and third nearest neighbors in Masiphumelele.

**Figure 4:**
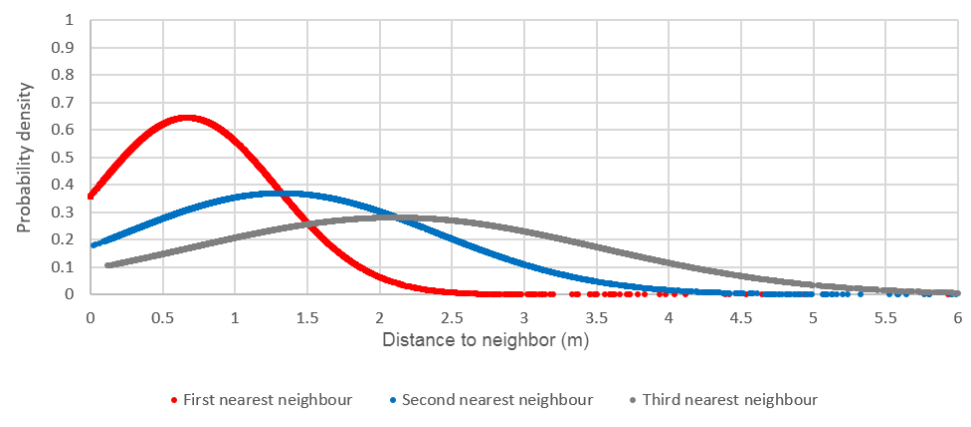
Normalized distribution of the distance between dwellings and their first, second, and third nearest neighbors in Klipfontein Glebe.

Calculating the distance between each dwelling and its first, second, and third nearest neighbors allows for a better understanding of density and separation distances within individual settlements. Examining the normalized distribution (Figures 3 and 4) can give an indication of the likelihood of a value on the x-axis occurring. The results show that the settlement of Masiphumelele has lower separation distances (small distance to first nearest neighbor) and is more dense (smaller distance to second and third nearest neighbors) with a high probability density (y-axis) of the distances (x-axis) being small. On the other hand, Klipfontein Glebe has larger distances to the first 3 nearest neighbors with a smaller probability indicating a more dispersed settlement. It should, however, be noted that where two or more dwellings’ roofs touched, the cluster of dwelling was digitized as a single dwelling; in reality, the graphs would be slightly skewed to the left (enhanced positive skew).

In Figure 3 it can be seen that Masiphumelele is a denser settlement than Klipfontein Glebe (Figure 4) with homes being built close together. The distance to the first nearest neighbor in Masiphumelele peaks at <0.5 m, the second nearest neighbor peaks at just less than 1 m, and the third nearest neighbor peaks at around 1.5 m. On the other hand, Klipfontein Glebe is a more dispersed settlement ,with the first nearest neighbor peaking at around 0.7 m, the second nearest neighbor peaking around 1.4 m, and the third nearest neighbor peaking just over 2 m. This analysis can be carried out for all informal settlements in Cape Town individually.

**Figure 5:**
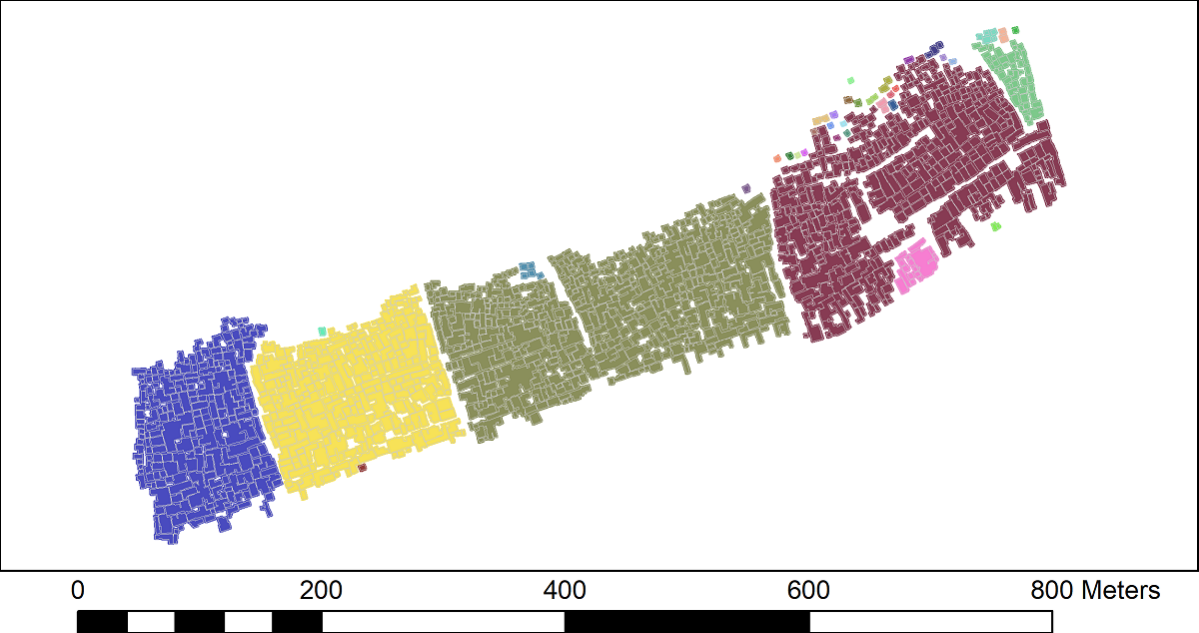
Clusters of dwellings in Masiphumelele that would need to self-isolate together. Different colors indicate group of dwellings that will be unable to practice social distancing from neighbors within the same color cluster.

**Figure 6:**
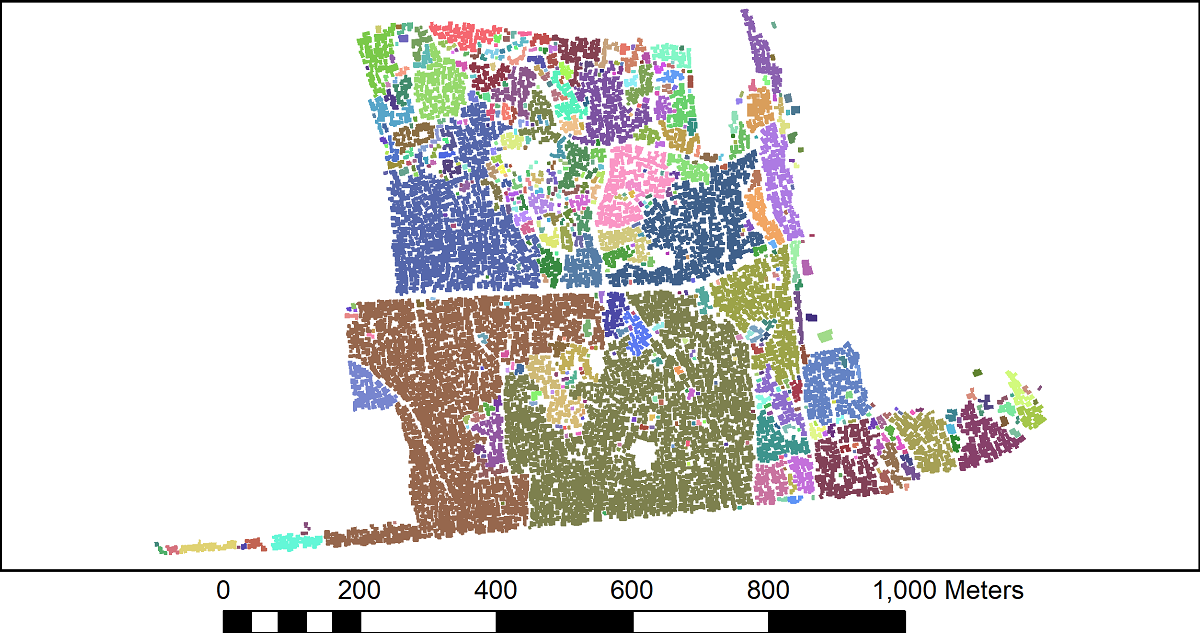
The clusters of dwellings in Klipfontein Glebe that would need to self-isolate together. Different colors indicate group of dwellings that will be unable to practice social distancing from neighbors within the same color cluster.

The results of buffering the dwelling outlines by 1 m are shown for Masiphumelele (Figure 5) and Klipfontein Glebe (Figure 6). It can clearly be seen that Masiphumelele poses a high risk for COVID-19 spread, as the groups of dwellings that would have to self-isolate together are typically large. The canals in Masiphumelele are effectively acting as breaks between dwellings, preventing even larger clusters.

For Klipfontein Glebe (Figure 6), the picture is more varied. There are some large clusters (for example, the bottom center olive green cluster) that represent a high transmission risk, but there are also smaller clusters throughout the settlement where the residents of these homes would be able to self-isolate with a smaller neighborhood.

**Figure 7:**
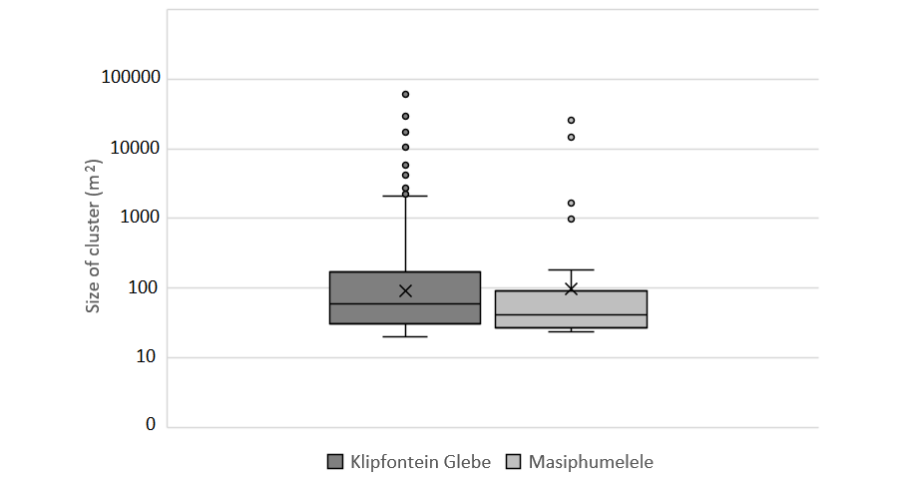
Box and whisker chart of size of clusters in Klipfontein Glebe and Masiphumelele. Note the log scale on the y-axis.

Descriptive statistics of the size of the clusters for Masiphumelele and Klipfontein Glebe (Table 1) show that the largest cluster occurred in Klipfontein Glebe; however, the mean value for Masiphumelele exceeded that of Klipfontein Glebe. The number of people living in these clusters is unknown, and it is unlikely that data at this level exists; however, a count of the dwellings is possible, and extrapolations about the estimated number of people per household can be made. The normalized distribution graphs (Figures 3 and 4) seem to show that Masiphumelele posed a higher risk with respect to the nearest neighbors than Klipfontein Glebe; however, the box and whisker chart in Figure 7 together with the descriptive statistics (Table 1) appear to paint a somewhat different picture. Klipfontein Glebe has the larger maximum cluster size and the larger median with a smaller standard deviation. However, the presence of some large clusters within the Masiphumelele data set, together with the lower count, result in a higher mean in Masiphumelele.

**Table 1:** Descriptive statistics of the size (m2) of clusters in Klipfontein Glebe and Masiphumelele.

Statistics	Klipfontein Glebe clusters	Masiphumelele clusters
Mean, m^2^	581	2432
Standard error, m^2^	164	1156
Median, m^2^	60	42
Standard deviation, m^2^	3999	7124
Minimum, m^2^	20	24
Maximum, m^2^	67312	30350
Number of clusters, n	593	38

## Discussion

### Principal Results

The results imply that social distancing (short of a lockdown) would be difficult to achieve in the two selected settlements. To effectively maintain social distancing, residents would, in effect, be unable to leave their homes. This is impractical, given that many homes are not serviced and lack toilets and running water. Even in the case of a complete lockdown (as is currently underway), residents would be asked to do the impossible, as they would be unable to leave their homes to access toilets and water while maintaining a safe 2-m separation distance. In addition, the living conditions inside homes are generally cramped and overcrowded with inadequate insulation, making staying indoors unbearably uncomfortable, particularly on hot days. Given the results of this paper, when implementing lockdowns, the authorities may need to take a more nuanced approach and consider implementing shut down at the community level, rather than at the household level.

The principal finding of this research is that, in the selected settlements, distance to each dwelling’s first 3 nearest neighbors illustrated that the settlement of Masiphumelele is constructed in a denser fashion when compared with the Klipfontein Glebe settlement, which, although some portions of the settlement are dense, is generally more dispersed. The first, second, and third nearest neighbors peak at approximately 0.5 m, 1.0 m, and 1.5 m, respectively, for Masiphumelele, and approximately 0.7 m, 1.4 m, and 2 m, respectively, for Klipfontein Glebe. This implies that implementing social distancing will likely be more challenging in Masiphumelele than in Klipfontein Glebe. However, using a 2-m social distancing measure, it was demonstrated that large portions of Klipfontein Glebe would also be unable to effectively implement social distancing.

### Limitations

A known limitation to this method is that many residents have to walk to a water stand and toilet, as many of the informal settlements are not serviced at the dwelling level. This creates unavoidable movement of people, and the pathways taken from dwellings to these communal points will be frequently used. Furthermore, these communal points will themselves be locations for potential disease spread, and, much like John Snow’s original research on epidemiology in 1854 [14], actions to prevent disease spread at these locations should be taken (although different from those implemented by Snow). Analysis of these data, if they indeed exist, should occur in parallel and in combination with the work presented here. Furthermore, it is reiterated that this method alone does not represent the entire picture of vulnerability to COVID-19 transmission in Cape Town informal settlements.

### Comparison to Prior Work

Similar work has not been found in the literature. Where vulnerability to COVID-19 or other disease has been mapped, it tends to consider data such as census data to identify density of populations, poverty indicators, and proportion of the population that fall in the vulnerable category [11]. Other uses of GIS in the COVID-19 pandemic has been widespread, mostly showing the location and magnitude of caseload or fatalities (a list is available on the website of the Center for Infectious Disease Research and Policy [15]).

### Conclusions

If the assumption presented earlier in the paper holds true, then effectively implementing social distancing in informal settlements in Cape Town will present a challenge. However, community containment poses its own challenges. Thus, containment of the spread of COVID-19 in Cape Town to prevent it reaching the informal settlements is likely to be a key consideration for authorities and decision makers. This will hold true for many other cities within Africa and the developing world, in general. However, data on informal settlements at the level that has been presented here is lacking in most, if not all, cities. Should the method presented here be deemed useful to decision makers, a mobilization of volunteer GISs as well as a machine learning approach would be proposed to produce the data required in the shortest a time period possible.

## Abbreviations

COVID-19: coronavirus disease
GIS: geographic information system
WHO: World Health Organization
